# Effect of Wheat Milling Process on the Distribution of *Alternaria* Toxins

**DOI:** 10.3390/toxins11030139

**Published:** 2019-03-01

**Authors:** Elizabet Janić Hajnal, Jasna Mastilović, Ferenc Bagi, Dejan Orčić, Dragana Budakov, Jovana Kos, Zagorka Savić

**Affiliations:** 1 Research Center for Technology of Plant Based Food Products, Institute of Food Technology, University of Novi Sad, 21000 Novi Sad, Serbia; jasna.mastilovic@fins.uns.ac.rs (J.M.); jovana.kos@fins.uns.ac.rs (J.K.); 2 Department of Plant and Environmental Protection, Faculty of Agriculture, University of Novi Sad, 21000 Novi Sad, Serbia; bagifer@polj.uns.ac.rs (F.B.); dbudakov@polj.uns.ac.rs (D.B.); zagorka.savic@yahoo.com (Z.S.); 3 Department of Chemistry, Biochemistry and Environmental Protection, Faculty of Sciences, University of Novi Sad, 21000 Novi Sad, Serbia; dejan.orcic@dh.uns.ac.rs

**Keywords:** wheat, milling process, alternariol, alternariol monomethyl ether, tenuazonic acid, LC-MS/MS

## Abstract

*Alternaria* toxins are mycotoxins produced by various *Alternaria* species which, besides the *Fusarium* species, represent the principal contaminants of wheat worldwide. As currently, only limited information on the behaviour of *Alternaria* toxins during processing of cereals is available, the objective of this study was to investigate the effect of the dry milling process of wheat on *Alternaria* toxins distribution. Alternariol (AOH), alternariol monomethyl ether (AME) and tenuazonic acid (TeA) content were analysed by high performance liquid chromatography coupled to tandem mass spectrometry (LC-MS/MS) in all milling fractions of untreated (control), fungicide-treated, *Alternaria tenuissima* inoculated and commercial wheat sample. After dry milling process, in last break and milling flows and by-products, increased concentration of examined *Alternaria* toxins was detected. TeA was quantified in almost all milling fractions in all tested wheat samples, while AOH and AME were detectable mostly in last break and milling flows and by-products. In respect to the contamination with *Alternaria* toxins, white flour can be considered as relatively safe product. Since *Alternaria* toxins are concentrated mainly in the peripheral parts of the kernel, a special attention should be given to their content in low-grade flours and milling by-products.

## 1. Introduction

Cereals and cereal by-products play an important role in the daily human diet, as well as in animal nutrition. Cereals are exposed to numerous biotic and abiotic stress factors during all phases, from cultivation to processing. The most common chemical contaminants of cereal crops are mycotoxins—secondary metabolites produced by toxigenic filamentous fungi as their natural protection either in the field or during storage [1]. Among cereals, wheat (*Tritricum aestivum*) is the second most produced grain worldwide [2,3] and the major share of produced wheat is subjected to the milling process that converts it to flour. Further, flour is processed into various foods such as breads, pasta, noodles and cakes, which represent a significant share of the human diet [4]. After harvest of wheat, as first-stage physical methods, cleaning and sorting processes are used to sort and clean grains based on kernel uniformity, weight, size and shape. These processes, by removing kernels with extensive mould growth, broken kernels, fine materials and dust, may reduce mycotoxins contamination in wheat; however, there is no step that destroys mycotoxins [5]. During the milling process, wheat grains are ground and separated into milling fractions based on particle size. Mycotoxins tend to be concentrated in the bran, flour shorts screenings and middlings (outer fractions) that are mainly used as animal feed, while in flour or semolina (inner fractions) intended for human consumption their concentrations are reduced [5]. Regarding wheat, the knowledge of mycotoxins distribution in milling fractions is mainly limited to *Fusarium* toxins, especially to deoxynivalenol (DON) [5]. Besides *Fusarium* spp., fungi of the genus *Alternaria* are considered as another group of ubiquitous pathogens on wheat leaves and ears. They are widespread in both humid and semi-arid regions and can infect growing plants in the field [6]. Among *Alternaria* species, especially *Alternaria (A.) alternata* and *A. tenuissima* are frequently associated with several plant diseases of small grain cereals (black point, kernel and leaf blight) [7,8,9,10] and both of them are capable to produce a variety of mycotoxins [8,9,10,11,12,13]. *Alternaria* toxins can be grouped into five different structural classes: dibenzopyrone derivatives—alternariol (AOH), alternariol monomethyl ether (AME) and altenuene (ALT); tetramic acid derivatives—tenuazonic acid (TeA) and iso-tenuazonic acid (iso-TeA); perylene derivatives—altertoxins I (ALX-I), altertoxin II (ALX-II) and alertoxin III (ALX-III) and stemphyltoxin III, a cyclic tetrapeptide—tentoxin (TEN) and *Alternaria alternata* f. sp. *lycopersici* toxins (AAL-toxins) [6]. The most common *Alternaria* toxins—AOH, AME, TeA and TEN—are generally found in certain grains and grain-based products, tomato and tomato products, sunflower seeds and sunflower oil, fruits and fruit products including fruit juices and in beer and wine [6]. Since these mycotoxins can be found in almost all food products, the continuous consumption of food contaminated by *Alternaria* toxins is an increasing concern for human health due to their possible harmful effects [14]. Some of *Alternaria* toxins have been described to possess genotoxic and mutagenic properties. In addition, they show cytotoxic, fetotoxic and/or teratogenic activity; they are mutagenic, clastogenic and oestrogenic in microbial and mammalian cell systems and they inhibit the cell proliferation [6]. Despite the fact that *Alternaria* toxins can be found in almost all food and feed products and that they might exhibit harmful effects on human and animal health, currently there are no specific international regulations or any national regulation in the world for any of the *Alternaria* toxins in food and feed, with the exception of Bavarian health and food safety authority who decided to limit the TeA content in sorghum/millet-based infant food at 500 µg/kg [15]. This is the first official decision of an authority regarding *Alternaria* toxins worldwide. In order to enable better understanding of the real risk for public health, opinions of different European scientific committees [6,16] recognized the need to collect more information about occurrence, toxicity and stability and fate of *Alternaria* toxins during storage and processing of food and feed. However, only limited information on the stability and fate of *Alternaria* toxins during storage and processing of food and feed is available [6] especially during processing of wheat. One of the few studies refers to investigation of the fate of AOH, AME and ALT under various baking conditions, using flour spiked with the toxins in the presence (wet baking) or absence (dry baking) of water [17]. After dry baking at 230 °C for 1 h, a pronounced degradation was achieved (90% for ALT, 70% for AOH and 50% for AME), while after wet baking conditions (for 45–60 min at 200 °C or for 30–45 min at 230 °C), no degradation of examined *Alternaria* toxins was observed [17]. In recently published study by Janić Hajnal et al. [18] the possibility of reduction of *Alternaria* toxins (AOH, AME and TeA) by extrusion processing of whole wheat flour was investigated. The highest reduction of all three *Alternaria* toxins was achieved when high raw material moisture (w = 24 g/100 g), high feeding rate (q = 25 kg/h) and medium screw speed (v = 390 rpm) were applied. Under these extrusion conditions, a reduction of 65.6, 87.9 and 94.5%, was achieved for TeA, AOH and AME, respectively.

To the best of our knowledge, there is no data about the fate of *Alternaria* toxins during wheat milling process. Therefore, the main objective of this study was to evaluate the effect of wheat milling process on the distribution of AOH, AME and TeA in processed wheat samples.

## 2. Results and Discussion

The initial concentration of AOH in an uncleaned commercial wheat sample was 1.67 µg × kg^−1^, while in other examined wheat samples AOH was absent, as can be seen from the Table 1. After cleaning, in this sample the content of AOH was reduced only by 25.6%. From the uncleaned wheat samples AME were quantified in commercial wheat sample, as well as in wheat samples inoculated with *A. tenuissima* with the initial concentration of 0.61 and 0.46 µg × kg^−1^, respectively. After cleaning, content of AME was below of limit of detection (LOD) in inoculated wheat sample, while in commercial wheat sample its content was reduced only by 8.2%. TeA was quantified in all the investigated samples and it was the most abundant of the monitored mycotoxins. After cleaning of the control, fungicide-treated, inoculated and commercial wheat grain samples, the content of TeA were reduced by 39.5, 40.7, 53.6 and 45.9%, respectively, in comparison to the initial TeA concentrations in the uncleaned wheat samples.

Although there are no published data regarding the effects of wheat cleaning process on the content of *Alternaria* toxins, the results obtained in this study are in agreement with the results published so far regarding effect of wheat cleaning process to decrease the content of present mycotoxins before milling, summarized by Schaarschmindt and Fauhl-Hassek [5] and confirm again the importance of cleaning process for obtaining relatively safe whole grain for milling.

In accordance with the objectives set out in this study, the cleaned samples of the control, fungicide-treated and *A. tenuissima* inoculated wheat, as well as commercial wheat, were ground in the pilot scale mill (Bühler MLU 202). From the point of the interpretation of the results regarding the presence of *Alternaria* toxins in milling fractions of wheat, it is important to look at the yield of the obtained fractions (Table 2).

**Figure 1:**
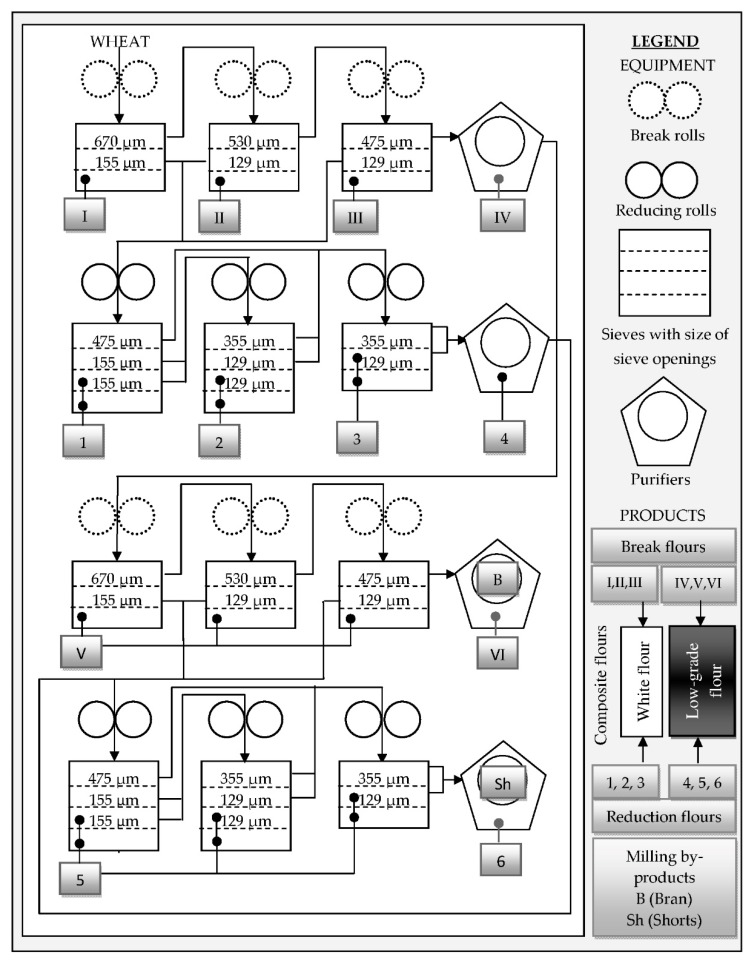
Simplified diagram of wheat milling with laboratory mill Bühler MLU 220.

Generally, for all tested samples, in accordance with the usual milling fraction ratios in the milling technology, the most common fractions are white flour fractions from the first milling runs (reduction flours 1, 2, 3). There is a significant share of the first two breaking fractions as well (break flours I, II), while the third breaking flour (break flour III) is represented with a smaller share. All these flours belong to a group of white flours (Figure 1), which mostly originate from the central parts of endosperm and contain a very small portion of the peripheral parts of the kernel.

Flours from the tail-end breaking runs (break flours IV, V, VI) and reducing runs (reduction flours 4, 5, 6) belong to darker flours which make up a composition of low-grade flour (Figure 1) with a higher content of minerals. Furthermore, dark flours contain larger portions of the kernel periphery which represent much lower shares in milling fractions, usually less than 2% of the total milling yield/extraction. As can be seen from the Table 2, milling by-products represent from 17.3 to 24.4% (bran and shorts fractions) of the total extraction with a dominant share of large (walloping) bran (14.1 to 15.1%).

The obtained milling fractions were analysed for *Alternaria* toxins content. Distribution of AOH, AME and TeA in milling fractions for all applied treatments is given in Table 1.

Only in commercial wheat sample concentration of AOH was above the LOD. Moreover, in the milling fractions obtained from all examined samples AOH was quantified in at least one of the fractions. The wheat milling fractions with the parts of wheat kernel where AOH is concentrated are primarily later breaking and reducing runs (tail-end passages), as well as milling by-products (shorts and bran). This result indicates that AOH is concentrated mainly in the peripheral parts of the kernel, which predominantly get into these fractions during the milling process.

In the initial wheat samples, AME was present only in commercial wheat sample. Despite this, similar to AOH, AME was found in certain milling fractions (Table 1) in all the samples tested. In the fractions of fungicide-treated wheat sample, AME was detected only in the last reducing (reduction flour 6) and breaking passages (break flour VI), indicating its presence in the peripheral parts of the kernel, which predominantly get into these fractions during the milling process. In the other flour samples, especially those originating from wheat samples with detectable amounts of AME, this toxin was, as a rule, quantified in all the milling by-products, in almost all the dark flours that originated from later breaking and reducing streams, again confirming the localization of mycotoxins in the peripheral parts of wheat kernels. It should be emphasized that AME was also found in certain light flours originated from the first breaking and reducing streams, which make up the largest part of the final milling products used for human consumption.

Particularly interesting results of toxin distribution in milling fractions (Table 1) were obtained in the case of TeA, which was present in all tested samples in significant quantities. First of all, it should be noted that significant differences in terms of the TeA content were observed in obtained flour fractions. Shorts and bran contained the highest amount of TeA, while high concentrations of TeA were observed in the last reducing (reduction flour 6) and breaking passages (break flour VI) in all cases. Therefore, including of milling fractions with a low TeA content, that is, the first fractions and rejecting of fractions with high toxin content in the composition of final products indicates the possibility of a technological reduction and control of TeA content in final milling products.

In order to determine and compare the significance of the influence of milling fraction and treatment in wheat production on registered levels of *Alternaria* toxins in flours and milling by-products a multi factorial analysis of variation was conducted (Table 3). Based on results obtained it can be concluded, that both investigated factors (wheat treatment (WT) and milling fraction (MF)) and their interaction (MT × MF) express significant influence on the level of *Alternaria* toxins content in wheat milling fractions.

The milling yields and AOH, AME and TeA mass balance of each milling product are reported in Table 4 to facilitate the comparison of the applied treatments for insight into the distribution of examined *Alternaria* toxins in the final milling products. The mass balance for each milling product (white flour, low-grade flour, shorts and bran) was calculated from the combination of the yield data during the milling process and the AOH, AME and TeA content. The white flours consist of the following milling fractions: reduction flours 1, 2, 3 and break flours I, II and III, while the composition of low-grade flours (dark flours) makes up reduction flours 4, 5, 6 and break flours IV, V and VI.

The milling yield of white flour (break flours I, II, III and reduction flours 1, 2, 3), as the main milling product of wheat for human consumption ranged from 66.9 to 75.1%, while for low-grade flour that is, dark flour (break flours IV, V, VI and reduction flours 4, 5, 6) the milling yield ranged from 7.4 to 8.7%. Regarding distribution of examined *Alternaria* toxins in white flour, absence of AOH was recorded in all cases, while AME was present only in white flour obtained from commercial wheat sample and its content amounted to 8.0% of the total contamination of the kernels. On the other hand, TeA was present in each of the white flour obtained and its content amounted from 7.65 (commercial wheat sample) to 19.8% (wheat sample inoculated whit *A. tenuissima*) of the total contamination of the kernel. However, the recorded concentration of AME and TeA in white flours intended for human consumption was relatively low. In case of contamination and distribution of examined *Alternaria* toxins in obtained low-grade flour (dark flour), it can be noted that only low-grade flour obtained from wheat samples which was treated with fungicide was not contaminated by AOH and AME. In other cases, content of AOH and AME amounted from 8 to 21.1% and from 8.72 to 64.2% of the total contamination of the kernel, respectively. The quantified concentration of these mycotoxins was low, while significantly higher content of TeA (37.0–73.8 µg × kg^−1^) was recorded in all low-grade flour obtained. Of the total contamination of the kernel, content of TeA in low-grade flours amounted from 9.7 (wheat treated with fungicide) to 10.9% (non-treated wheat sample). Since, low-grade flour (dark flour) which mainly contained end-tail breaking fractions (break flours IV, V, VI) and reducing fractions (reduction flours 4, 5, 6) in which the content of TeA is higher (Table 1) in relation to the initial reducing runs (reduction flours 1, 2, 3) and breaking runs (break flours I, II, III), a great attention should be given related TeA content in these milling product even in the case of relatively low initial content in wheat.

Regarding distribution of the analysed *Alternaria* toxins in the milling by-products, it can be noted from the Table 4 that in bran AOH and TeA were present with the highest content, which amounted from 55.6 to 83.8% and from 49.7 to 66.1% of the total contamination of the kernel, respectively. Regarding the distribution of AME, the same situation was observed in bran samples which were obtained from naturally contaminated wheat samples (non-treated and commercial), while in the bran samples which originated from wheat treated with pesticide and wheat inoculated with *A. tenuissima* the largest share of AME was found in shorts and its content amounted 100 and 41.9% of the total contamination of the kernel, respectively. Milling by-products are very often used for animal feed but can also be used for human consumption. Therefore, great attention should be given to the fact that *Alternaria* toxins are concentrated in these fractions of the milling process. The obtained results in this study could not be completely compared to the published data, since to the best of authors’ knowledge there is no previously published study regarding the fate of *Alternaria* toxins during dry milling process of wheat and their distribution in obtained milling fractions. However, comparing the results obtained in this study with the distribution of DON, the most studied regulated mycotoxin in wheat-based foods during dry milling process (obtained in a pilot mill and by industrial milling), summarized by Schaarschmindt and Fauhl-Hassek [5], the similar rule can be noted. Namely, the published data have shown that lower levels of DON are found in the main products, that is, white flour derived from the endosperm, whereas higher DON concentrations are present in the bran-containing by-products compared to the initial level in whole grains. The results obtained in this study indicate that during dry milling process distribution of examined *Alternaria* toxins takes place according to the same principle as in the case of DON.

Furthermore, there is also a lack of data on the presence of *Alternaria* toxins in commercial milling products. One of the few studies refers to the presence of *Alternaria* toxins (AOH, AME, TeA and TEN) in samples of commercial wheat flour from China [19]. Among 181 analysed wheat flour samples 6.1, 91.2, 99.4 and 97.2% were contaminated with AOH, AME, TeA and TEN, respectively. Relatively high mean values for TeA (88.4 µg × kg^−1^), AOH (30.2 µg × kg^−1^) and AME (27.1 µg × kg^−1^) were observed in these wheat flour samples, while the maximum content of AOH, AME, TeA and TEN were 98.7, 61.8, 520 and 129 µg × kg^−1^, respectively. Furthermore, currently published scientific report related to the dietary exposure assessment to *Alternaria* toxins in the European population [20] indicated that the most common *Alternaria* toxins (AOH, AME, TeA and TEN) are present in unspecified wheat milling products. According this scientific report among mentioned *Alternaria* toxins, TeA is the most frequent contaminant in commercial wheat milling products and this toxin is present with higher content in relation to others *Alternaria* toxins. Further, as already mentioned, published data around the world (summarized by EFSA [6] and published data in the period 2012–2018 from Norway [21], Germany [22], Serbia [23], China [19] and Albania [24]) indicate frequent contamination of wheat with *Alternaria* toxins, so their presence is certainly expected in milling products of wheat. Namely, the predominant *Alternaria* toxins in wheat samples from Norway were AOH and AME (100% each), followed by ALT-I (96.4%) and TeA (21.4%), while the highest median (116 µg × kg^−1^) and maximum content (305 µg × kg^−1^) were observed for AOH [21]. Among 1064 analysed wheat samples from Germany, 30.3% was contaminated with TeA, with maximum quantified content of 4224 µg × kg^−1^, while AOH and AME rarely occurred in German wheat. ALT was present only in 7 out 267 wheat samples from Germany [22]. Regarding contamination of Serbian wheat, among 92 analysed wheat samples during the period of three years (2011–2013), 68.5% were contaminated with TeA, 12.0% with AOH and 6.5% with AME. The maximum and mean toxin concentrations were 2676 and 92.4 μg × kg^−1^, 48.9 and 18.6 μg × kg^−1^ and 70.2 and 39.0 μg × kg^−1^ for TeA, AOH and AME, respectively [23]. TeA was also a predominant *Alternaria* toxin in terms of either frequency or concentration in wheat from China and it was detected in 100% (370/370) samples with the highest mean (289 µg × kg^−1^) and maximum concentration (3330.7 µg × kg^−1^). Further, wheat from China were frequently contaminated with TEN (77%), followed by AOH (47%) and AME (15.1%). It should be noted, that TEN occurred with high mean (43.8 µg × kg^−1^) and maximum content (258.6 µg × kg^−1^) in Chinese wheat, also. Among 71 harvested wheat samples (2014–2015) from Albania, 82.9% in 2014 and 86.1% in 2015 were contaminated with some of the investigated Alternaria toxins (AOH, AME, TeA and TEN) and the major detected *Alternaria* toxin was TeA with maximum, mean and median concentration of 175.7, 29.9 and 16.5 µg × kg^−1^, respectively [24]. According to the published data, it could be noted, that *Alternaria* toxins are frequently detected in wheat and therefore there is a great possibility for their presence in wheat-based product.

## 3. Conclusions

Based on results obtained it can be concluded that both investigated factors (initial level of toxins in wheat as a consequence of treatments in production and milling fraction) and their interaction express significant influence on the level of *Alternaria* toxins content in wheat milling fractions. In wheat milling process, even for wheat with concentration of *Alternaria* toxins under the LOD of the analytical method used, increased concentration of *Alternaria* toxins can be expected in fraction containing peripheral kernel parts, including last break and milling flows and by-products. In white flours, which represent the main part of the final products of wheat milling, the level of TeA was reduced by (83.6 to 90.4%) of the initial concentration in wheat before cleaning. In relation to TeA concentration in wheat after cleaning, in white flours the level of TeA was reduced by 72.3 to 82.3%. Therefore, white flour can be considered relatively safe product in respect to the contamination with TeA. In the by-products of wheat milling (bran and shorts), TeA was present in 2 to 4 times and in 3 to 6 times higher concentrations than in uncleaned and cleaned wheat, respectively. The wheat milling fractions with the parts of wheat kernel where AOH and AME were concentrated were primarily later breaking and reducing runs (tail-end passages), as well as milling by-products (shorts and bran). This result indicates that AOH and AME were also concentrated mainly in the peripheral parts of the kernel, which predominantly get into these fractions during the milling process. The future research should be related to the behaviour of *Alternaria* toxins under industrial conditions, in terms of managing the milling process of wheat in order to obtain safe final milling products. In addition, results of this first report may also represent useful support in the future consideration of the legal maximum limit for most common *Alternaria* toxins.

## 4. Materials and Methods

### 4.1. Samples

The presented research represents one segment of comprehensive research targeted at investigations of incidence and possibilities of reduction of *Alternaria* toxins in wheat and wheat processing products. Experiment was carried out in the 2012/2013 season on the wheat (*Triticum aestivum* cv. Sirtaki). Samples of (1) wheat treated by fungicide,(2) wheat inoculated by *A. tenuissima* (isolate code: SOR13IIZA1 from the fungal collection of Faculty of Agriculture, Novi Sad derived from naturally infected kernels of winter wheat in North-Eastern part of the Republic of Serbia in which *Alternaria* species were previously identified by morphological characteristics according to Simmons [25].) and (3) non-treated (control) wheat were harvested in quantity of about 60 kg of each. Obtained samples, used for present investigation, were also used to examine the possible protective effect of the hulls on the content of *Alternaria* toxins in wheat (results already published [26]). Further, in the same published paper authors in detail described weather conditions during the period of flowering of wheat (May 2013), as well as the procedure of multiplication of *A. tenuissima* isolates, wheat inoculation on the field (100 m^2^) and collection of wheat samples, which were also used in this investigation. Furthermore, (4) commercial wheat sample with visible discoloration called black points, which indicate potential fungal infestation by *Alternaria* spp., was procured (about 60 kg) from the warehouse for this study. The batches of all four wheat samples were well mixed in a NAUTA mixer (model 19387, maximal capacity 25 kg per batch, Haarlem, The Netherlands) before taking samples for analysis and before dry milling process. Mixing homogeneity of wheat samples was assured by taking of four subsamples for analysis of investigated *Alternaria* toxins levels before cleaning of each examined wheat sample.

Wheat samples (500 g) were ground to a 1 mm particle size using a laboratory mill (KnifetecTM 1095 mill, Foss, Hoganas, Sweden) to obtain the milled whole wheat from uncleaned and cleaned wheat samples.

After cleaning of 10 kg of each wheat sample by a Laboratory Grain Cleaner 5150 (Huddinge, Sweden), a portion of 3 kg was milled using a Bühler MLU 202 (Bühler, Uzwil, Switzerland) pilot scale mill. The wheat preparation for milling included wheat grain tempering (to ensure differences in the structural characteristics between the anatomic parts of the wheat kernel) to 13.5% of moisture with a holding period of 24 h. This was followed by the second tempering up to the moisture content in wheat grain of 15% with an additional holding period of 30 min. Milling was carried out according to an extended scheme, presented in Figure 1. Milling of wheat grains is a gradual reduction process consisting of sequential and consecutive size reduction (roller mills) and separation (plansifters). The process involves breaking open the kernel, scraping the endosperm from the bran (break rolls made corrugated cast steel) and gradually reducing the chunks of endosperm into flour by smooth reducing rolls. Segregation between the kernel parts occurs in plansifters (using sieves with different sieve openings), where sieves separate particles of different size and in purifiers, where sieves and air-flow separate particles of different size, specific gravity and shape. Generally, in the milling process through consequent milling and sieving operations numerous flour fractions, originating dominantly from different part of wheat kernel, are obtained and subsequently mixed in order to obtain composite flours of desired quality and composition. According to presented milling diagram (Figure 1) from each wheat sample 12 milling stream flours (6 break flours (I–VI) and 6 reduction flours (1–6)) and milling by-products (shorts and bran) were obtained.

Each of the obtained milling fraction of each wheat sample were separately collected, weighed and kept at −20 °C before examination.

### 4.2. Moisture Content

Moisture content in wheat samples and in all milling fractions was determined according to ISO 712 [27] and was expressed on the dry basis.

### 4.3. Sample Preparation

The modified method from Siegel et al. [17], described in our previous study [23], was used for sample preparation. Briefly, approximately 1 g (exact weights known) of homogenized samples was mixed with 7 mL of water. Subsequently, 2 mL of 2 mol/L aq. HCl and 5 mL of ethyl acetate (EtOAc) were added and shaken (Orbital Shaker PSU-10i, BOECO, Hamburg, Germany) for 45 min, ultrasonicated for 10 min (ATM40-3LCD, Madrid, Spain) and shaken again for 45 min. The extract was transferred into plastic cuvettes and centrifuged (Centrifuge 5804 R, Eppendorf, Germany) at 5000 rpm for 15 min to achieve complete phase separation. Thereafter, 2 mL of the upper (EtOAc) layer was transferred into glass cuvette and evaporated under a stream of nitrogen (Reacti-Therm I#18821, Thermo Scientific, Bellefonte, PA, USA). The dry residue was dissolved in 1 mL of LC-MS-grade methanol (MeOH) and transferred to an HPLC vial through an Econofilter PTFE (13 mm, 0.2 μm) syringe filter (Agilent Technologies, Beijing, China) and stored at −20 °C until analysis.

### 4.4. Instrumental Conditions

*Alternaria* toxins (AOH, AME, TeA) were quantified by high performance liquid chromatography coupled to tandem mass spectrometry (LC-MS/MS) using our previously published method without any modifications, including the equipment and materials [23].

### 4.5. Method Validation

The method was validated by in-house quality control procedure following the guidelines of Commission Decision EC 657/2002 [28]. Method validation was performed in terms of matrix effects, linearity, trueness, precision, limit of detection (LOD) and limit of quantification (LOQ). Method validation, as well as method validation data for wheat samples, was previously described in detail by Janić Hajnal et al. [23]. Method validation for milling fractions included determination of LOD and LOQ (Table S1). For linearity studies, the calibration curves for all of the compounds in pure solvent (solvent calibration, SC) and in matrix (matrix-matched calibration, MMC) were obtained by plotting the peak areas against the concentrations of the corresponding calibration standards at five calibration levels in the ranges present in Table S1 for AOH, AME and TeA, respectively. The linearity of calibration curves was expressed by the correlation coefficient (*r*^2^). To differentiate between extraction efficiency and matrix-induced signal suppression/enhancement, the slope ratios of the linear calibration functions were calculated to yield the apparent recovery (*R_A_*), that is, the overall method recovery and the signal suppression/enhancement (*SSE*) due to matrix effects. The recovery of the extraction step (*R_E_*), that is, sample preparation recovery, was calculated by dividing the overall recovery by the matrix effect as follows:

*R_A_* (%) = 100 × *a_SP_*/*a_S_* (1)

*SSE* (%) = 100 × *a_MMC_*/*a_SC_* (2)

*R_E_* (%) = 100 × *a_SP_*/*a_MMC_* (3)

where *a_MMC_* is the slope of matrix-matched calibration, *a_SC_* is the slope of solvent calibration and *a_SP_* is the slope of spiked sample-prepared curve. For overall method recovery assessment, the wheat flour fractions were spiked in triplicate, over the ranges present in Table S1 for TeA, AOH and AME (five-point *R_A_*), respectively. Spiked samples were left overnight at room temperature to allow solvent evaporation and equilibration between analytes and matrix and were analysed using matrix-matched calibration curve. For the matrix-matched calibration curves (MMC), the wheat flour fractions were enriched with working standard solutions at the final reconstitution step, confirming linearity over the range presented in Table S1 for TeA, AOH and AME (five-point MMC), respectively. The calibration curve in pure solvent (five-point SC) was prepared over the same range as well as for *R_A_* and MMC for all of the compounds and was used for matrix effects evaluation.

The precision of the method was expressed in terms of repeatability, that is, as the relative standard deviation (%RSD) of 6 replicates at three concentration levels (Table S2) using the spiked wheat flour fractions and the MMC curve.

### 4.6. Alternaria Toxins Determination

In order to eliminate the effect of matrix, *Alternaria* toxins were quantified by external matrix-matched calibration procedure. Two matrix-matched calibration curves were constructed for TeA in the concentration range from LOD to 100 µg × kg^−1^ and from 100 to 250 µg × kg^−1^. For AOH and AME, one calibration curve was constructed in the concentration range from LOD to 10 µg × kg^−1^ and from LOD to 5 µg × kg^−1^, respectively. Linearity testing gave values of correlation coefficients (*r*^2^) above 0.9910 in all the investigated ranges. The obtained results were corrected for sample preparation recovery (*R_E_*) and were expressed on a dry matter basis. All samples were prepared and analysed in triplicates.

Further, based on the obtained data for weight of each flour fraction and its AOH, AME and TeA content, according to the scheme for obtaining of composite white and low-grade flours provided in Figure 1, *Alternaria* toxins content in these composite flours, as the products that would be actually placed at the market by the mills, was calculated according to the following equation:

*Alternaria* toxin content in composite flour = Σm_i_ × T_i_/Σm_i_ (4)

where m_i_ weight (g) of the fractions that make up the composition of white and low-grade flours; T_i_ *Alternaria* toxins (AOH, AME, TeA,) content (µg × kg^−1^) in the fractions that make up the composition of white and low-grade flours.

### 4.7. Statistical Analysis

One way and multifactorial analysis of variance of obtained data were carried out. For determination of significance of differences among the mean values Duncan’s multiple comparison tests was used. For not detectable values (under LOD) of AOH and AME, data were adopted at the level of half of LOD. STATISTICA software v13.3 was used [29]. *P* values <0.05 were regarded as significant.

## Figures and Tables

**Table 1:** Content of *Alternaria* toxins in wheat samples and in their products of milling process.

Product		Non-Treated Wheat Sample			Sample of Wheat Treated with Fungicide			Sample of Wheat Inoculated with *Alternaria tenuissima*			Commercial Wheat Sample		
		Mycotoxin Content (µg × kg^−1^)											
		AOH	AME	TeA	AOH	AME	TeA	AOH	AME	TeA	AOH	AME	TeA
**Uncleaned grain**		<LOD	<LOD	59.5 ^B^	<LOD	<LOD	30.7 ^A^	<LOD	0.46	96.6 ^D^	1.67	0.61	78.07 ^C^
**Cleaned grain**		<LOD	<LOD	36.0 ^B^	<LOD	<LOD	18.2 ^A^	<LOD	<LOD	44.8 ^D^	1.26	0.56	42.26 ^C^
**Break flours**	I	<LOD	<LOD	4.63 ^a^	<LOD	<LOD	3.12 ^a^	<LOD	<LOD	13.40 ^ab^	<LOD	<LOD	4.98 ^a^
	II	<LOD	0.17 ^ab^	9.39 ^b^	<LOD	<LOD	6.02 ^a^	<LOD	0.20 ^a^	10.43 ^a^	<LOD	0.30 ^a^	29.66 ^b^
	III	<LOD	<LOD	17.70 ^c^	<LOD	<LOD	15.88 ^c^	<LOD	<LOD	58.55 ^e^	1.78 ^ab^	0.47 ^b^	56.26 ^c^
	IV	4.29 ^c^	2.15 ^f^	52.78 ^f^	<LOD	<LOD	47.64 ^f^	5.43 ^c^	1.53 ^d^	98.87 ^g^	2.71 ^bc^	0.60 ^b^	80.42 ^d^
	V	<LOD	0.24 ^b^	28.84 ^d^	<LOD	<LOD	29.24 ^e^	<LOD	0.48 ^b^	40.99 ^d^	<LOD	0.85 ^c^	51.69 ^c^
	VI	5.49 ^d^	1.01 ^d^	56.10 ^f^	0.86 ^b^	0.15 ^a^	52.80 ^g^	7.15 ^d^	2.04 ^e^	64.91 ^e^	4.68 ^d^	2.21 ^e^	169.41 ^g^
**Reduction flours**	1	<LOD	<LOD	4.88 ^a^	<LOD	<LOD	3.22 ^a^	<LOD	<LOD	6.46 ^a^	<LOD	0.12 ^a^	2.54 ^a^
	2	<LOD	<LOD	4.08 ^a^	<LOD	<LOD	3.44 ^a^	<LOD	<LOD	9.24 ^a^	<LOD	<LOD	3.60 ^a^
	3	<LOD	<LOD	12.83 ^b^	<LOD	<LOD	10.27 ^b^	<LOD	0.43 ^b^	17.61 ^b^	<LOD	0.17 ^a^	5.02 ^a^
	4	2.07 ^b^	1.54 ^e^	21.31 ^c^	<LOD	<LOD	25.00 ^d^	<LOD	0.50 ^b^	24.72 ^c^	2.45 ^bc^	0.51 ^b^	34.74 ^b^
	5	<LOD	0.83 ^c^	47.05 ^e^	<LOD	<LOD	30.51 ^e^	<LOD	0.69 ^c^	37.71 ^d^	3.83 ^cd^	1.07 ^d^	112.18 ^e^
	6	8.24 ^e^	3.60 ^g^	86.70 ^g^	0.87 ^b^	0.17 ^b^	60.20 ^h^	3.37 ^b^	0.87 ^c^	90.81 ^f^	6.34 ^e^	2.10 ^e^	153.13 ^f^
**Bran**		4.36 ^c^	0.30 ^b^	145.14 ^h^	3.18 ^c^	<LOD	131.20 ^j^	7.47 ^d^	0.40 ^b^	159.58 ^h^	11.16 ^f^	2.56 ^f^	215.41 ^h^
**Shorts**		5.23 ^d^	0.70 ^c^	154.81 ^i^	3.24 ^c^	0.30 ^c^	110.51 ^i^	9.67 ^e^	2.67 ^f^	193.71 ^i^	11.89 ^f^	3.95 ^g^	231.44 ^i^

Different letters (A, B, C, D) for the same mycotoxin indicate significant differences (*p* < 0.05) between values according to the Duncan’s multiple range test within the wheat samples. Different letters (a, b, c, d, e, f, g, h, i, j) for the same mycotoxin indicate significant differences (*p* < 0.05) between values according to the Duncan’s multiple range test within each of the wheat sample. LOD: limit of detection (µg × kg^−1^); AOH: alternariol; AME: alternariol monomethyl ether; TeA: tenuazonic acid; I, II, III, IV, V, VI: break flours; 1, 2, 3, 4, 5, 6: reduction flours.

**Table 2:** Yield of the obtained milling fractions and its moisture content of examined wheat samples.

Yield of the Wheat Fractions (%)/Moisture (%)					
Fraction		Non-Treated Wheat sSample	Sample of Wheat Treated with Fungicide	Sample of Wheat Inoculated with *Alternaria tenuissima*	Commercial Wheat Sample
**Break flours**	I	5.0/11.76	6.9/12.45	5.8/13.01	3.3/11.95
	II	11.3/13.06	11.1/13.77	11.1/13.28	6.9/12.86
	III	1.4/11.15	1.5/11.88	1.3/10.52	1.2/11.20
	IV	1.6/13.16	1.7/14.01	1.4/13.19	1.3/13.86
	V	1.3/10.07	1.6/9.73	1.2/9.25	1.4/10.32
	VI	0.9/11.28	0.9/12.08	0.8/11.01	0.6/11.28
**Reduction flours**	1	16.9/12.96	17.5/13.71	20.3/12.98	14.4/13.03
	2	26.9/12.87	26.1/13.28	27.2/12.83	23.5/13.21
	3	11.5/11.62	11.5/12.65	9.4/11.90	17.5/12.54
	4	1.9/11.79	1.9/11.32	2.0/11.19	3.5/12.01
	5	2.3/9.95	1.5/10.47	1.3/10.22	1.2/10.33
	6	0.8/9.60	0.6/9.51	0.7/9.78	0.7/10.10
**Bran**		14.7/11.76	14.5/12.46	14.1/13.13	15.1/12.18
**Shorts**		3.5/9.98	2.8/10.21	3.4/9.98	9.3/10.29

Abbreviations: milling fraction designations I, II, III, IV, V, VI: break flours; 1, 2, 3, 4, 5, 6: reduction flours.

**Table 3:** Multifactorial analysis of variance corresponding to the content of Alternariol (AOH), Alternariol monomethyl ether (AME) and tenuazonic acid (TeA).

	dF	SS	MS	F	p
**AOH**					
**WT**	3	142.07	47.36	183.48	0.00
**MF**	13	1034.12	79.55	308.21	0.00
**WT × MF**	39	363.20	9.31	36.08	0.00
**Error**	112	28.91	0.2581		
**AME**					
**WT**	3	20.33	6.78	872.68	0.00
**MF**	13	59.43	4.57	588.81	0.00
**WT × MF**	39	60.56	1.55	199.99	0.00
**Error**	112	0.8696	0.0078		
**TeA**					
**WT**	3	47152.5	15717.5	1232.03	0.00
**MF**	13	490509.0	37731.5	2957.63	0.00
**WT × MF**	39	57249.8	1467.9	115.07	0.00
**Error**	112	1428.82	12.76		

dF: Degrees of freedom; SS: Sum of squares; MS: Mean square; WT: Wheat treatment; MF: Milling fraction; AOH: alternariol; AME: alternariol monomethyl ether, TeA: tenuazonic acid.

**Table 4:** Milling properties of wheat samples and distribution of AOH, AME and TeA.

	Yield (%)	Concentration (µg × kg^−1^)			Mass Balance **			Distribution (%) ***		
		AOH	AME	TeA	AOH	AME	TeA	AOH	AME	TeA
**Non-Treated (control) Wheat Sample**										
**Cleaned wheat**		<LOD	<LOD	36.0						
White flour *	73.1	<LOD	<LOD	6.67	0.00	0.00	4.94	0.00	0.00	13.9
Low-grade flour *	8.7	2.51	1.41	44.4	0.22	0.12	3.89	21.1	64.2	10.9
**Bran**	14.7	4.36	0.30	145.1	0.64	0.04	21.3	61.6	23.3	60.1
**Shorts**	3.5	5.23	0.70	154.8	0.18	0.02	5.35	17.3	12.6	15.1
**Total**	100				1.04	0.19	35.5	100	100	100
**Wheat Sample Treated with Fungicide**										
**Cleaned wheat**		<LOD	<LOD	18.2						
White flour *	74.6	<LOD	<LOD	5.05	0.00	0.00	3.77	0.00	0.00	13.1
Low-grade flour *	8.1	<LOD	<LOD	37.0	0.00	0.00	2.99	0.00	0.00	10.3
**Bran**	14.5	3.18	<LOD	131	0.46	0.00	19.1	83.8	0.00	66.1
**Shorts**	2.8	3.24	0.30	111	0.09	0.008	3.04	16.2	100	10.5
**Total**	100				0.55	0.008	28.9	100	100	100
**Sample of Wheat Inoculated with *Alternaria tenuissima***										
**Cleaned wheat**		<LOD	<LOD	44.8						
White flour *	75.1	<LOD	<LOD	10.9	0.00	0.00	8.19	0.00	0.00	19.8
Low-grade flour *	7.4	2.12	0.93	54.2	0.16	0.07	4.00	10.2	31.5	9.7
**Bran**	14.1	7.47	0.40	160	1.06	0.06	22.6	68.6	26.3	54.6
**Shorts**	3.4	9.67	2.67	194	0.33	0.09	6.57	21.3	41.9	15.9
**Total**	100				1.54	0.22	41.3	100	100	100
**Commercial Wheat Sample**										
**Cleaned wheat**		1.26	0.56	42.3						
White flour *	66.9	<LOD	0.11	7.48	0.00	0.07	5.00	0.00	8.00	7.65
Low-grade flour *	8.7	2.78	0.90	73.8	0.24	0.08	6.44	8.00	8.72	9.85
**Bran**	15.1	11.2	2.56	215	1.69	0.39	32.5	55.6	42.8	49.7
**Shorts**	9.26	11.9	3.95	231	1.10	0.37	21.4	36.4	40.5	32.8
**Total**	100				3.03	0.90	65.4	100.0	100.0	100

* Values are calculated (see Section 4.6); ** Calculated by (Yield (%) × Concentration (µg × kg^−1^)); *** Calculated as the quantity expressed in percentage of *Alternaria* toxins in each milling product; LOD: limit of detection; AOH: alternariol; AME: alternariol monomethyl ether, TeA: tenuazonic acid.

## Supplementary Materials

The following are available online at https://www.mdpi.com/2072-6651/11/3/139/s1. Table S1: Recovery data of the employed analytical method based on solvent (*R_A_*) and matrix-matched (*R_E_*) calibration curves and matrix effect (SSE), limit of detection (LOD) and limit of quantification (LOQ), Table S2: Repeatability of alternariol (AOH), alternariol monomethyl ether (AME) and tenuazonic acid (TeA).
