# Supplementary material for: Effect of Wheat Milling Process on the Distribution of Alternaria Toxins
Source: Toxins (Basel). 2019 Mar 1;11(3):139. doi: 10.3390/toxins11030139 (PMC6468426; doi:10.3390/toxins11030139)
Supplement: Supplementary file 1 [file toxins-11-00139-s001.pdf]

Supplementary Materials

Table S1. Recovery data of the employed analytical method based on solvent (R_A) and matrix-matched (R_E) calibration curves and matrix effect (SSE), limit of detection (LOD) and limit of quantification (LOQ).

Fractions	Spiking level* (μg × kg^{-1})			Overall method recovery R_A (%)			Sample preparation recovery R_E (%)			Matrix effect SSE (%)			LOD/LOQ (μg × kg^{-1})		
	**AOH**	**AME**	**TeA**	**AOH**	**AME**	**TeA**	**AOH**	**AME**	**TeA**	**AOH**	**AME**	**TeA**	**AOH**	**AME**	**TeA**
**I**	0.5–10	0.1–5	5–30	97.3	80.1	84.0	81.0	98.9	105.6	120.0	81.1	79.5	0.5/1.65	0.3/1.0	2.5/7.5
**II**	0.5–10	0.1–5	2.5–30	87.2	75.5	84.9	119.7	86.2	74.5	123.3	87.6	114	0.5/1.65	0.3/1.0	1.5/5.0
**III**	0.5–10	0.1–5	2.5–50	127.7	71.4	83.2	95.6	85.4	81.3	133.6	83.7	102.3	0.5/1.65	0.1/0.3	1.5/5.0
**IV**	0.5–10	0.1–5	7.5–100	126.9	44.6	110.6	86.4	79.8	89.6	146.8	55.8	123.5	0.75/2.5	0.3/1.0	2.5/7.5
**V**	0.75–10	0.1–5	2.5–50	85.2	89.0	84.4	94.5	97.7	90.2	90.2	91.2	93.6	0.75/2.5	0.1/0.3	1.5/5.0
**VI**	0.5–10	0.1–5	7.5–200	80.5	63.8	57.4	103.1	82.6	77.8	128.1	77.3	73.9	0.5/1.65	0.1/0.3	1.5/5.0
**1**	0.5–10	0.1–5	2.5–15	77.6	57.4	72.3	100	82.0	87.2	77.6	70.0	82.9	0.75/2.5	0.1/0.3	1.5/5.0
**2**	0.5–5	0.5–5	2.5–10	76.9	93.0	81.8	94.4	109.9	74.6	81.4	118.2	109.7	0.5/1.65	0.3/1.0	1.5/5.0
**3**	0.5–10	0.1–5	2.5–15	99.4	77.5	101.3	96.4	81.1	91.5	103.1	95.5	110.8	0.5/1.65	0.1/0.3	1.5/5.0
**4**	0.5–10	0.1–5	7.5–50	84.0	57.6	74.9	102.1	75.0	109.8	82.2	76.6	68.2	0.75/2.5	0.3/1.0	1.5/5.0
**5**	0.75–10	0.1–5	7.5–100	91.4	94.1	98.9	72.8	72.9	89.9	122.3	129.2	110.1	0.3/1.0	0.1/0.3	1.5/5.0
**6**	0.5–10	0.1–5	7.5–200	76.3	73.3	63.7	85.3	92.3	91.2	89.4	79.4	69.8	0.5/1.65	0.1/0.3	1.5/5.0
**Bran**	0.75–10	0.1–5	7.5–200	83.1	67.5	83.8	78.8	102.7	90.5	105.6	65.7	92.6	0.75/2.5	0.3/1.0	1.5/5.0
**Shorts**	0.75–10	0.1–5	7.5–200	55.1	63.2	71.2	70.2	85.3	81.9	78.4	74.1	86.9	0.5/1.65	0.3/1.0	1.5/5.0

AOH: alternariol; AME: alternariol monomethyl ether; TeA: tenuazonic acid.

Table S2. Repeatability of alternariol (AOH), alternariol monomethyl ether (AME) and tenuazonic acid (TeA).

Fractions	Repeatability (n = 6) RSD (%)					
	Spiking level (μg × kg⁻¹)	AOH	Spiking level (μg × kg⁻¹)	AME	Spiking level (μg × kg⁻¹)	TeA
I	0.75	11.8	0.3	14.3	5	16.9
	2.5	9.5	0.75	13.2	10	11.3
	5	8.3	2.5	12.2	15	6.3
II	0.75	10.4	0.1	19.4	5	13.7
	2.5	9.0	0.3	15.0	15	10.8
	5	7.2	0.75	13.6	30	5.3
III	0.75	13.5	0.1	14.9	7.5	12.3
	2.5	9.3	0.3	13.1	15	10.5
	5	5.8	0.75	12.4	30	8.2
IV	2.5	18.1	0.3	14.1	10	7.5
	5	13.6	0.75	9.8	50	3.7
	7.5	8.2	2.5	8.8	100	3.3
V	0.75	17.9	0.3	18.0	10	18.3
	2.5	13.9	0.75	15.9	30	15.3
	5	11.7	2.5	9.3	50	11.0
VI	0.75	14.0	0.1	12.4	30	4.8
	2.5	13.4	0.75	5.8	100	4.1
	7.5	9.0	2.5	4.2	200	3.5
1	0.75	11.8	0.1	17.3	2.5	12.2
	2.5	10.4	0.3	12.3	5	10.3
	5	6.0	0.75	11.4	10	7.5
2	0.5	17.9	0.5	12.8	2.5	16.8
	2.5	13.1	2.5	10.8	7.5	15.3
	5	6.9	5	8.8	10	14.9
3	0.75	17.6	0.1	18.2	5	7.1
	2.5	17.1	0.3	12.1	10	6.6
	5	11.5	0.75	11.0	15	5.9
4	0.75	15.4	0.3	16.9	2.5	9.4
	2.5	10.7	0.75	14.7	5	8.7
	5	5.8	2.5	10.5	15	5.5
5	0.75	17.3	0.3	13.8	30	13.9
	2.5	11.0	0.75	12.7	50	9.7
	5	9.1	2.5	11.4	100	9.3
6	0.75	18.3	0.1	12.0	50	8.5

	2.5	15.6	0.75	9.6	100	1.2
	5	11.6	2.5	3.6	200	1.0
	2.5	15.4	0.3	18.9	50	17.1
**Bran**	5	10.2	0.75	15.4	100	14.8
	10	8.2	2.5	12.1	200	5.7
	2.5	16.1	0.75	16.5	50	11.4
**Shorts**	5	12.7	2.5	12.7	100	11.1
	10	9.8	5	12.5	200	8.3
